# Objective and Subjective Factors Influencing Breast Reconstruction Decision-Making After Breast Cancer Surgery: A Systematic Review

**DOI:** 10.3390/healthcare13111307

**Published:** 2025-05-30

**Authors:** Valentini Bochtsou, Eleni I. Effraimidou, Maria Samakouri, Spyridon Plakias, Aikaterini Arvaniti

**Affiliations:** 1 Department of Psychiatry, Faculty of Medicine, Democritus University of Thrace, 68100 Alexandroupolis, Greece; msamakou@med.duth.gr (M.S.); aarvanit@med.duth.gr (A.A.); 2 First Surgical Department, Faculty of Medicine, University Hospital of Alexandroupolis, Democritus University of Thrace, Dragana, 68100 Alexandroupolis, Greece; eeffraem@med.duth.gr; 3 Department of Physical Education and Sports, University of Thessaly, 42100 Trikala, Greece; spyros_plakias@yahoo.gr

**Keywords:** breast cancer, mastectomy, breast reconstruction, decision-making, healthcare disparities, patient-centered care

## Abstract

**Background/Objectives**: Breast reconstruction (BR) following mastectomy plays a critical role in post-cancer care by offering both physical and psychological benefits. Despite advancements in techniques and shared decision-making (SDM), BR uptake remains inconsistent. This systematic review aims to synthesize evidence on objective (medical and socioeconomic) and subjective (psychological and personal) factors influencing BR decision-making among women undergoing mastectomy for breast cancer. **Methods**: A systematic search was conducted across PubMed, ScienceDirect, OVID, and Google Scholar, identifying peer-reviewed studies published between January 2013 and 25 July 2024. Eligible studies examined determinants of BR decisions in women undergoing therapeutic mastectomy, excluding perspectives of non-patient stakeholders and post-decision outcomes. The risk of bias and study quality were assessed using the Quality Appraisal for Diverse Studies (QuADS) tool. This review was registered in PROSPERO (CRD42023456198) and followed PRISMA guidelines. **Results**: Twenty-seven studies comprising 994,528 participants across 16 countries met the inclusion criteria. The objective factors included age, comorbidities, insurance coverage, physician recommendations, and healthcare access. The subjective factors encompassed body image concerns, self-esteem, fear of recurrence, and emotional readiness. Younger age, private insurance, and active physician counseling were associated with increased BR uptake, while older age, lack of information, and financial or logistical barriers reduced uptake. Regional disparities were noted across healthcare systems. **Conclusions**: BR decisions are influenced by complex, interrelated clinical, psychological, and systemic factors. Integrating SDM tools, enhancing patient education, and addressing healthcare inequities are essential for supporting informed and equitable BR decision-making. Future research should prioritize longitudinal studies and policy interventions to improve access to and patient satisfaction with BR outcomes.

## 1. Introduction

Breast cancer remains a significant global health issue, representing approximately 12% of all newly diagnosed cancer cases and ranking as the most common malignancy among women [1]. Despite advancements in detection and treatment, mastectomy remains a central therapeutic approach for many patients. The consequences of mastectomy extend beyond oncological outcomes, significantly influencing body image, self-esteem, and overall quality of life (QoL) [2].

Recent studies emphasize that the loss of a breast following mastectomy can result in profound psychosocial distress, impacting emotional stability, social functioning, and sexual well-being [3,4]. Women frequently report experiences of altered body image, reduced femininity, stigma, and emotional isolation, which can persist for years after treatment if unaddressed [2,5]. In particular, breast absence has been associated with higher rates of depression, anxiety, and dissatisfaction with the overall cancer survivorship experience [2]. Accordingly, restoring body image has become a pivotal element of comprehensive post-mastectomy care, leading to the emergence of breast reconstruction (BR) as a critical intervention offering aesthetic and psychological benefits that enhance long-term well-being [6,7].

Despite the availability of reconstructive techniques, the global uptake of BR remains suboptimal, with notable disparities in access, decision-making, and utilization [5]. Utilization rates are significantly higher in high-income countries, where access to trained surgical teams, healthcare infrastructure, and insurance coverage is more robust [8]. Conversely, women in lower-resource settings often face limitations related to affordability, health literacy, and logistical constraints [9]. Socioeconomic status, education, and insurance coverage are major determinants of access to BR, with marginalized groups experiencing significantly lower rates of uptake and completion [10,11,12]. Age disparities persist as well, with older women being less likely to undergo BR despite comparable safety outcomes [10].

Breast reconstruction can be categorized by timing into immediate, delayed, or delayed–immediate procedures. Immediate BR (IBR), performed concurrently with mastectomy, provides prompt restoration of body contour and potential psychosocial benefits. IBR may not be suitable in certain clinical contexts, such as the anticipated need for post-mastectomy radiation therapy (PMRT) or when patients face decisional uncertainty at the time of diagnosis [13]. Delayed BR (DBR), typically performed after the completion of adjuvant treatments, remains a viable alternative but may be influenced by diminished patient motivation or limited institutional resources [14]. The delayed–immediate BR approach, which involves temporary tissue expansion followed by definitive reconstruction after PMRT, balances oncologic safety and aesthetic planning, providing flexibility when postoperative treatment needs are uncertain [15,16]. However, medical contraindications, such as poorly controlled comorbidities, smoking, or obesity, may preclude reconstruction or limit the choice of technique, especially in immediate settings [13,17,18].

Non-clinical factors also substantially influence BR decisions. Guidance from healthcare providers, peer experiences, and social support networks are among the most frequently cited influences [19,20,21,22,23]. Models of shared decision-making (SDM), especially those incorporating digital decision aids and structured counseling, have been shown to improve patient satisfaction and confidence [19,24,25]. Nonetheless, disparities persist due to inconsistent communication, clinician-related biases, and cultural perceptions [19].

Previous research has identified the determinants of BR decision-making, pointing to the interplay between socioeconomic status, healthcare access, racial disparities, and psychosocial considerations, such as body image and femininity [26,27,28,29]. Flitcroft et al. [6] emphasized the importance of patient-centered approaches that align treatment options with individual values while addressing structural barriers.

Given the multifactorial nature of BR decision-making, an updated synthesis of the recent literature (2013–July 2024) is essential to comprehensively understand the determinants shaping patient choices. Given the changing landscape of cancer care and the persistent inequities in BR access, a contemporary evaluation of the factors influencing women’s choices is needed.

This systematic review aims to comprehensively synthesize the objective (medical and socioeconomic) and subjective (psychological and personal) factors that shape BR decision-making among women undergoing mastectomy for breast cancer. By understanding how systemic and clinical determinants interact with psychological influences, this study seeks to inform patient-centered care strategies and policy development. Ultimately, it aims to promote equitable access to BR and support informed, person-centered decision-making across diverse healthcare settings.

## 2. Materials and Methods

### 2.1. Protocol and Registration

**Figure 1:**
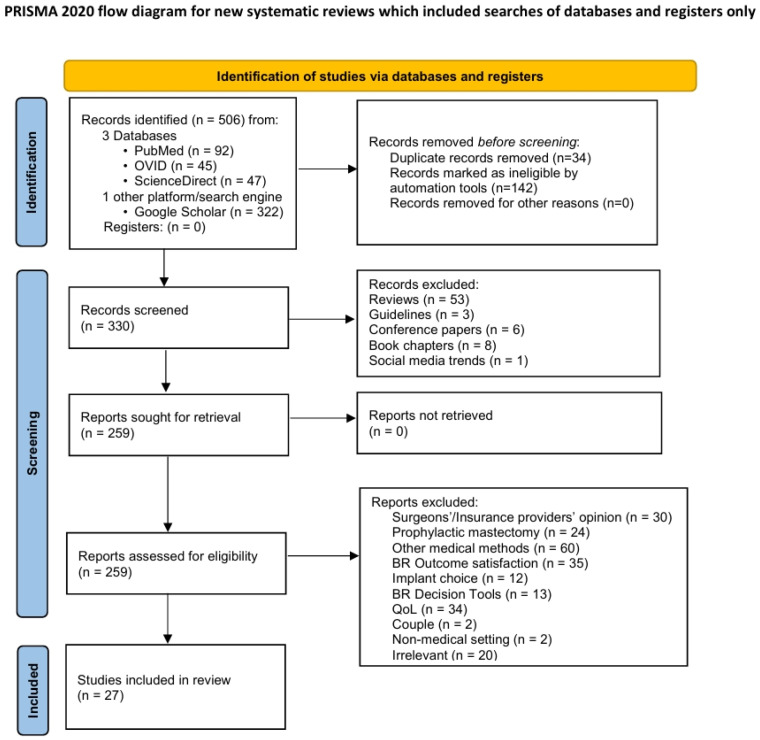
Prisma flow diagram.

This systematic review was conducted and reported according to the Preferred Reporting Items for Systematic Reviews and Meta-Analysis (PRISMA) guidelines to ensure a comprehensive evaluation of the literature [30]. Τhe research stages followed by the researchers are presented in Figure 1. This review was registered with the PROSPERO protocol registration number CRD42023456198.

### 2.2. Search Strategy

A systematic search was conducted across the following databases: PubMed, ScienceDirect, and OVID. Google Scholar was also used as a supplementary source to identify potentially relevant peer-reviewed articles not captured through traditional databases. The searches were conducted using structured keyword combinations, and the results were screened systematically, with only peer-reviewed journal articles considered for inclusion. The following keywords were used: breast cancer OR ductal carcinoma in situ OR mastectomy NOT prophylactic AND breast reconstruction AND influencing factors AND decision-making.

The time frame (2013–July 2024) was selected to capture studies reflecting contemporary clinical practices, advances in surgical techniques, and the increasing integration of shared decision-making in BR care [6,16,25]. Since 2013, growing attention has been paid to patient-centered outcomes, healthcare disparities, and the expansion of insurance coverage and guidelines supporting BR [11,31]. This period ensured methodological relevance and alignment with evolving BR policies and patient involvement models. The endpoint, 25 July 2024, corresponded to the final date of the literature search.

### 2.3. Selection Criteria

Only original articles published in peer-reviewed journals were included if they met the following criteria: (i) written in English (only English-language articles were included to ensure consistent interpretation of culturally sensitive decision-making factors and due to the lack of translation resources); (ii) published from 2013 to 25 July 2024 (last search date); (iii) focused on factors influencing BR decision-making; (iv) included a sample of women undergoing mastectomy after a breast cancer diagnosis (excluding prophylactic mastectomy, as decision-making in these cases occurs in a non-urgent, risk-based context, distinct from the time-sensitive, emotionally complex decisions faced by women undergoing mastectomy following a cancer diagnosis); (v) investigated only the feelings and opinions of women regarding BR (excluding perspectives from insurance officers, doctors, spouses, partners, or other relatives); (vi) focused on women who had not yet made a decision about BR (excluding studies on BR outcome satisfaction, BR type choice, BR decision tool use satisfaction, or quality of life changes post-BR); and (vii) conducted in a medical setting.

Articles were excluded if they (i) had been published in other sources (books, conference special issues, social media, etc.) to ensure methodological consistency and adequate reporting standards, and only peer-reviewed journal articles were eligible, as they allowed for a uniform quality appraisal; (ii) were published outside the specified time frame (pre-2013 or after 25 July 2024); (iii) were written in languages other than English; (iv) focused on prophylactic mastectomy; (v) investigated perspectives other than those of the women undergoing BR (e.g., insurance officers, doctors, or family members); and (vi) examined post-decision outcomes, such as BR satisfaction, type choice, or QoL changes after BR.

Studies employing qualitative, quantitative, or mixed methods were included, provided they fulfilled all the predetermined inclusion and exclusion criteria.

### 2.4. Study Quality Assessment

The quality of the included studies was assessed using the Quality Assessment for Diverse Studies (QuADS) tool, which was developed to appraise methodological and reporting quality across studies with heterogeneous designs. QuADS includes 13 criteria that assess clarity of aims, rationale for design, data collection and analysis methods, integration of findings, reflexivity, and relevance to review objectives. Each criterion is scored on a 4-point scale (0–3), allowing for structured assessment across qualitative, quantitative, and mixed-methods studies. The tool has demonstrated strong inter-rater reliability and face and content validity in health services research contexts [32].

### 2.5. Data Synthesis and Analysis

Due to the heterogeneity of study designs, populations, and reported outcomes, it was not feasible to conduct a meta-analysis. Instead, a narrative synthesis approach was used to analyze and interpret the data. The synthesis followed three main steps: (1) extracting key information from each study regarding demographic, clinical, psychological, and systemic factors influencing BR decision-making; (2) organizing findings into two main categories—objective and subjective factors; and (3) summarizing and comparing patterns across studies to identify recurring themes, contextual variations, and inconsistencies in reported outcomes. Where available and informative, we also reported basic descriptive statistics (e.g., frequencies, percentages) from individual studies to support interpretation.

These analytical steps were discussed extensively among all co-authors to ensure shared understanding, enhance transparency, and minimize interpretive bias. The aim of the final synthesis was to provide a comprehensive overview of the current evidence on factors influencing BR decision-making across diverse healthcare and cultural contexts.

### 2.6. Study Design

#### Identification and Screening

Two authors (V.B. and A.A.) independently examined the titles and abstracts of conceivably relevant articles. Duplicate records were eliminated, and articles not meeting the predefined inclusion criteria were excluded. In cases of ambiguity, both reviewers conducted a full-text evaluation to ascertain eligibility.

A total of 506 publications were identified, of which 27 met the eligibility criteria for inclusion in this study. All types of reviews were independently screened and retrieved for reference purposes; however, none of the selected publications in this review were directly derived from them.

## 3. Results

### 3.1. Characteristics of Studies Included

Supplementary Table S1 provides an overview of the 27 included studies, including the study aim and timeframe, recruitment and study setting, number of participants and participation rate, design/methods, tools used, limitations/bias, reasons, findings, conclusions, and the overall quality score. Supplementary Table S2 provides a summary of the fundamental clinical information (cancer-, treatment-, and BR-related) extracted from all 27 included studies. Eight studies were from the US, four from Saudi Arabia, four from Australia, two from the Netherlands, and one each from New Zealand, Japan, Singapore, India, Poland, Hungary, France, Sweden, and the UK.

Although the selected articles were published from 2013 onward, the studies that they reported were conducted between 1998 and March 2022. The total number of participants was 994.528, ranging from 23 to 638.772 per study. The majority of participants were aged 50 years and older, with several studies reporting an overrepresentation of individuals in the 50–60 age range and a notable proportion exceeding 60 years.

The quality scores of the included studies, ranging from 31 to 38 out of a possible 39, indicated a consistently high methodological standard, with all studies demonstrating strong adherence to quality criteria, as assessed by the QuADS (Supplementary Table S3).

The 27 studies analyzed employed a variety of research designs, reflecting the methodological diversity used to explore BR decision-making and utilization. Cross-sectional studies were the most prevalent [3,8,9,22,23,24,33,34,35,36], often utilizing questionnaires and surveys to assess patient preferences, knowledge, and attitudes at a single time point. Retrospective cohort studies [20,37,38,39,40,41,42,43] were drawn from medical records and registry data, allowing for the examination of long-term BR trends and demographic influences over extended periods. Prospective studies [21,44,45,46] offered a forward-looking perspective, following patients over time to assess how their knowledge, psychosocial factors, and clinical circumstances impacted BR decisions, thereby providing stronger evidence for causal relationships. Qualitative studies [6,19,38,47] explored the lived experiences, emotional considerations, and cultural influences underlying patient choices, adding rich, patient-centered insights often missed by quantitative methods. Notably, population-based studies [20,31,48] utilized national or regional data registries to evaluate large-scale patterns in BR utilization and disparities across different demographic groups. Among the collection, one case–control study compared patients with and without BR to identify significant predictors and barriers [41]. Collectively, this methodological spectrum highlights the importance of using diverse study designs to comprehensively understand BR decision-making, combining statistical rigor with patient-centered perspectives.

### 3.2. Demographic and Clinical Information

Supplementary Table S1 provides information on population demographics, and Supplementary Table S2 summarizes the information about the cancer type/stage, time since mastectomy, BR type and timing, type of breast cancer treatment, and type and timing of surgery. The type/stage of cancer was not reported in eight studies. Where specified, the cancer stage/type ranged from 0 (ductal carcinoma in situ) to IV (metastatic breast cancer).

### 3.3. Factors Influencing BR

#### 3.3.1. Objective Factors

1. Age

Age appeared to be a key factor in the BR process, as mentioned in the majority of the reviewed articles (85%; 23 out of 27 articles). An important finding is that age affects the desire and willingness to undergo BR [9,23,33,37,39]. Some researchers have mentioned age as a predictor of BR [9,31]. Furthermore, four articles reported that BR was significantly correlated with younger age [37,38,40,43], while eight articles cited it in reverse, stating that older age was correlated with lower BR rates [20,24,31,36,38,41,42,46] or even no BR (nBR) [19,49]. One of these studies [46] also connected older age with poorer communication in BR. Only one of the reviewed articles found no significant differences between the BR and nBR groups when correlated with age [35]. The choice of the BR subtype has been discussed in several articles with variable findings. Sergesketter et al. mentioned that women closer to 45 years of age preferred tissue-based BR [20], a finding also confirmed by Mátrai et al., who added significant associations between age and women’s concerns as well as BR timing [23]. Danko et al., however, found that younger-aged women preferred implant-based BR vs. tissue-based and combined BR [48]. Filipe et al. reported that while some patients receiving IBR were older, overall, increasing age was independently associated with a significantly lower likelihood of undergoing immediate reconstruction after mastectomy [8]. Although age is an objective factor, three studies reported that age could be subjectively perceived by women [3,19] as well as surgeons [34], influencing BR decision-making and mainly leading to refusal.

2. Socioeconomic reasons

This category included socioeconomic status, employment/profession, income, and insurance. Twelve of the reviewed articles mentioned socioeconomic reasons as predictive [23,31,48], determining [20,24,37,42,50], or influencing other aspects of the decision-making procedure, such as information receipt [46], financial constraints/unaffordability [22,35,36], and reimbursement [36]. State insurance or no insurance at all were mentioned as negative BR predictors [20,48], whereas private insurance was associated with higher BR rates [24,37,42]. Two studies reported that women with higher incomes had higher odds of undergoing BR [31,46]. Unaffordability was mentioned by one out of five women opting out of BR in two studies [24,36]. Ng et al. did not agree with that finding, reporting that “high out-of-pocket cost was not a major reason for women selecting nBR” [40]. Miseré et al. mentioned that financial reasons did not play a substantial role in decision-making, clarifying that the Dutch state and private insurance companies fully reimbursed the procedure [38]. Nair et al. reported that almost one out of three women in their sample in India would opt for BR regardless of financial issues [22].

3. BR awareness and source of information

In total, 48% of the articles (13/27) referred to the importance of BR awareness for willingness to undergo the procedure. Three studies reported that preoperative information had a statistically significant impact on undergoing BR or declared interest in it [24,31,39]. One study mentioned it reversely, as a lack of information can be a barrier to BR, noting that misconceptions can negatively affect BR choices [8]. The most influential sources of information appear to be plastic surgeons and breast surgeons [22,24,33,45], breast cancer nurses [36], peers (women who have already undergone BR) [21,23], and the internet [23,24,40,51]. One study underlined the importance of videos and photos in decision-making [19]. Another study found that information about the BR option was significantly lower in older women, non-native English speakers, Asian or Latina women, underweight women, and women without private insurance [46].

4. Medical reasons

In total, 48% of the studies mentioned the relevance of medical issues (breast cancer treatment-related or other comorbid conditions) to BR decisions. Two studies [33,34] stated that women deciding to undergo BR were medically free of comorbidities and had no chronic illnesses compared with those who chose not to proceed with BR [46]. Concerning breast cancer (BC) itself, numerous researchers agree that an ascending stage, invasiveness, and lymph node involvement negatively influence BR decisions [8,20,31,34,35,47,48]. BC treatment has also been reported as a significant factor in women who underwent adjuvant treatment or no chemotherapy and are more receptive to BR [35,41,42].

5. Physician recommendation/influence

Surgeons were among the most common sources of information about BR [33,47]. Referring to physicians significantly influences BR consideration [21,33]. Breast and plastic surgeons were identified as the most influential figures in the BR decision-making process [21]. According to Blackmore et al. [19], 65% of women who considered breast surgeons as key influencers proceeded with BR, while 76% of those who regarded plastic surgeons as influential also underwent the procedure. An earlier study by Manne et al., however, reported that a consultation with a plastic surgeon did not significantly deteriorate anxiety and decisional conflict, according to their findings [45].

6. Role of partner

One study reported cohabitation status as a factor correlated with BR receipt, with 81% of partnered individuals choosing to have BR and 73% opting for IBR [49]. Nair et al. reported that 22% of the women mentioned that their decision was affected by their spouse wanting BR [22]. Two studies referred to positive relationship support as being associated with an increased likelihood of pursuing BR, with higher rates appearing in married women [20]. Blackmore et al., however, did not find a similar statistically significant association, merely that lower home/work responsibilities increased the BR likelihood [19]. Duggal et al. reported that only a quarter of the women in their survey were concerned about their partner’s satisfaction when deciding on BR [21]. Moreover, Manne et al. [45] did not find data supporting that the role of a partner is of key importance in BR decision-making. Nozawa et al. found that partner influence was particularly significant for unmarried women, as their decision regarding BR was more likely to be shaped by their partner’s perspective compared with those who were married or in long-term relationships [35].

7. Education

Six studies reported findings on the association between educational levels and BR procedures. First, according to Aljaaly et al., educational level affects BR awareness, which is a key issue in the whole procedure [33]. The higher the educational level, the higher the rates of BR awareness coming from social media. Three studies [38,39,48] found that a higher educational level is linked to the likelihood of (or even predicts) BR receipt. Awan et al. reported that education is a significant predictor of the desire to utilize BR [9]. Specifically, women with high or even low educational attainment were 21.65 and 7.99 times more likely to desire BR compared with women with no education, respectively [9]. One study reported no significant difference between the BR and nBR groups concerning educational level [35].

8. Race

White was the only race-related factor found to be predictive of IBR receipt compared with tissue-based and combined BR [48]. Caucasian origin, compared with Asian, was reported to have higher odds of performing post-mastectomy BR [42]. In a large sample of 346.418 patients in the US, women undergoing BR were less likely to be non-Hispanic Black or Hispanic [20]. Two studies found no significant association between race and BR decisions [43,46].

#### 3.3.2. Subjective Factors

1. Body image and self-esteem

The concepts of body image and self-esteem were reported in multiple studies and were closely associated with BR decisions. Two studies found a statistically significant association between the decision to undergo BR and the importance placed on body image by women [33,40]. The ability to wear/look good in clothes [21,39,40], as well as the subjective feeling of femininity [19,21,23,39,45], were reported as influencing factors. Women with stronger beliefs in favor of physical appearance restoration were more likely to choose BR [21,22,35,41,45,49]. Furthermore, women connecting BR with an improved body image also connected BR with higher self-confidence, linking physical wholeness to psychological wholeness [21,23,35,38,39,41,44,45,49].

2. Fear of recurrence

Another significant reason connected mostly with BR refusal was fear of cancer recurrence, the detection of which could reportedly be “masked” by BR [9,35,39,40,41,49]. Only one study reported on cancer recurrence oppositely: Guest et al. reported that a small number of women reported wanting BR to reduce the risk of cancer [44].

3. Concerns about additional surgery/hospitalization

A common reason for nBR, reported in seven studies, was the concern about additional surgery and/or hospitalization. Some reported that women were unwilling to undergo additional surgery [3,19,36]. Furthermore, the rest of the studies described an additional worry about risks, potential complications, and long-term problems of the BR procedure and, therefore, repeated hospitalizations [9,24,41,45].

Supplementary Tables S4 and S5 summarize the factors influencing BR choices, as discussed in the 27 articles included in this review.

## 4. Discussion

Breast reconstruction after mastectomy is a complex and multifaceted decision that can be influenced by several medical, psychological, social, and systemic factors [9,12]. This systematic review synthesized the existing literature to identify the key determinants that shape BR decision-making, highlighting both pragmatic and objective factors (e.g., demographic, clinical, and economic factors) and endogenous, individual characteristics (e.g., body image, personal preferences, and emotional well-being). Understanding these factors is vital to improving patient-centered care, ensuring informed decision-making, and addressing inequalities in access to and use of BR. While this review focused on personal, social, and systemic influences on BR decision-making, it is important to recognize that medical eligibility remains a foundational constraint. Clinical contraindications—such as uncontrolled comorbidities, high surgical risk, or smoking—may override patient preference and limit reconstruction options, particularly for immediate procedures [13,17,18].

The influencing factors were grouped into two overarching categories to interpret the findings: objective factors—such as age, health status, and socioeconomic conditions—that are externally observable and often influence eligibility or access, and subjective factors, which include internal, psychological, and emotional considerations, such as body image, self-esteem, fear of recurrence, and personal priorities. This framework allowed for a clearer understanding of how both measurable and experiential dimensions shape BR decisions in diverse healthcare contexts.

There is an ongoing discussion within publications about the role of age in BR decision-making. Age, on one hand, is subjectively perceived by women. In simple terms, how old a woman may feel and what other subjective needs she has may be associated with her perceived age (in terms of, e.g., body image, femininity, and self-esteem) [19,51]. On the other hand, from an objective, realistic point of view, chronological age assessed by surgeons or other healthcare or health insurance providers in relation to comorbidities influences the likelihood of proposing BR [21,34,36,46]. Women aged over 45 years tend to think about BR significantly less, according to several studies included in this review [9,21,23,33,37,39,40,41,42]. As Somogyi et al. state, “…the odds of having BR decrease by 3% for every year of increasing age…” [24]. Younger patients may be more likely to consider BR due to longer life expectancies than older women and potential concerns about body image [38]. They are mainly reported to select IBR [20,23,31,48]. Older women, however, either know immediately that they do not desire to proceed with BR [21] or may be “deprived” of the chance to receive information and discuss BR as much despite having an equal right to, regardless of their wishes and preference, due to ageism [7,21,52]. As suggested by Chang-Azancot et al., ageism in BR decision-making may contribute to the lower rates of BR in older populations, even though evidence indicates that surgical complications do not necessarily increase with age unless combined with other risk factors, such as radiotherapy or smoking [18]. Therefore, older women have equal rights to an informed BR choice without predetermining that they are not interested in BR, their body image is not a priority issue, or they are not healthy enough to undergo such a procedure [41,53,54,55]. Only one study reported a relatively high percentage (47%) of women older than 60 years who received DBR or IBR despite the finding that nBR was correlated with older age [49].

Surgeons and healthcare providers play a pivotal role in BR decision-making, with plastic and breast surgeons considered the most influential sources of information [19]. However, the extent of their influence varies, and in some cases, consultations with plastic surgeons do not significantly alleviate decisional conflict [45]. The lack of standardized counseling approaches may contribute to disparities in BR uptake, particularly among older women and socioeconomically disadvantaged groups [56]. Additionally, while surgeon recommendations are crucial, SDM models have emerged as an effective approach for ensuring patient autonomy and improving satisfaction [25]. The integration of SDM, including the use of decision aids, such as printed booklets, web-based tools, SDM aids, mobile applications, and visual decision aids, could help bridge existing knowledge gaps and facilitate more informed and patient-centered choices [6].

Socioeconomic factors play a crucial role in BR decision-making, influencing both accessibility and patient choices across different healthcare systems. Access to healthcare, insurance coverage, and financial resources can significantly impact a patient’s ability and willingness to undergo BR, even in developed countries with high standards of living [48]. In privatized healthcare systems, such as the US, higher income and private insurance are strong predictors of BR uptake, with privately insured women being significantly more likely to undergo the procedure than those relying on public or no insurance [11,48]. Regional differences should also be considered. While financial concerns serve as a major deterrent in countries with privatized healthcare, studies from nations with universal healthcare coverage indicate that BR rates are influenced more by awareness and physician recommendation than by financial constraints [8]. Financial barriers are less pronounced in European nations such as the Netherlands and Sweden, but disparities persist in awareness, physician counseling, and patient involvement in decision-making [8,38,56]. Meanwhile, countries with lower SESs, particularly in Eastern and Southern Europe, face additional challenges, such as limited access to trained plastic surgeons, lower BR awareness, and bureaucratic obstacles despite theoretical reimbursement coverage. Studies from Poland and Hungary highlighted that although BR is included in national healthcare systems, practical barriers, including a shortage of specialized professionals, hinder accessibility [23,36]. In Japan and India, cultural attitudes further shape decision-making, with financial concerns and limited discussions about BR contributing to lower uptake rates [22,35]. These cross-country variations highlight the need for tailored patient education programs and healthcare policies that address region-specific barriers, ensuring equitable opportunities for BR access and global decision-making.

In general, patients who were interested in undergoing BR were healthier and medically free of chronic illnesses [33,34,37,46,50]. Patients at different stages of breast cancer may have varying treatment priorities, impacting their decisions regarding BR [35]. Patients who require PMRT as part of their breast cancer treatment might have limitations on the timing and type of BR [20,33,50,57]. Patients who have plans for future treatments, such as chemotherapy, may prioritize information on therapy and coordinate the timing of BR accordingly [20,35,41,58]. The tumor grade and stage of disease were reported in some studies as influencing the BR decision in terms of type and timing [8,20,31,37,42,48], whereas Sue et al. did not confirm this in their findings [43]. Nodal involvement also decreased the likelihood of BR after mastectomy [34,48,56]. Moreover, a patient’s overall health and medical history may influence their eligibility for different types of BR procedures. Factors such as body type, smoking habits, and general health can impact the suitability of certain reconstruction methods [37,46], consistent with the existing literature [17,59].

BR awareness is crucial for SDM. In other words, access to information about BR options, benefits, risks, and potential outcomes can influence a patient’s decision about BR. Demographic aspects such as increased age or low education levels are linked to limited BR information attainment [46]. Online resources and social media lead information provision, followed by breast cancer team professionals, peers, and relatives [19,22,23,33,36,39,47]. However, misinformation can be an insurmountable barrier in the BR decision-making process [4,60]. Plastic surgeons are mentioned as the most influential healthcare specialists in BR decision-making, leading discussions along with surgical oncologists toward informed decisions [61]. This role is well understood in the US [45], while challenges remain about the equal dissemination of critical information regarding BR. Peers [21], women who have undergone a BR, appear to play a particularly promising role in awareness issues before mastectomy by taking part in preoperative discussions about BR with their contribution “based on experience” [62]. Somogyi et al. mentioned that such discussions significantly increase the odds of opting for BR [24].

Educational level was not reported as a significant predictive factor in most studies. Nevertheless, Danko et al. noted in their retrospective analysis of 638.772 medical records in the US that completion of high school was a predictive factor for BR receipt [48], which is understandable, considering the amount of information a woman must process to come to her decision [63]. Education level and BR were significant predictors of BR in publications from Central European (Hungary) and Middle Eastern countries, such as Saudi Arabia [9,23,33,39]. Higher educational attainment was associated with a higher rate of BR awareness derived from social media and the internet [33]. Generally, the adaptation of available BR information is needed, according to different educational levels, health literacy, and the desire to be involved in the decision-making process, as well as proper care that BR information material is provided rapidly and comprehensively [31,62,63].

Most women in the reviewed studies decided about BR autonomously, regardless of their partners’ preferences. Opinions of others about BR seemed to be of secondary importance. Positive relationship support [20], including acceptance of a mastectomy scar or a flat hemithorax, seemed to play a more significant role in empowering women to make the most suitable decision according to their desires [36]. Unmarried women are more likely to have a partner’s influence [35]. A low percentage of women (approximately 20%) mentioned decisive influence in Nair et al.’s study in a relatively small sample in India [22], while a similar percentage in Duggal et al.’s work (USA) reported concern about their partner’s satisfaction with the BR outcome when deciding on it [21]. The recent literature on the subject highlights partners’ equal need for BR information and the importance of their participation in the BR decision-making process [64,65]. The availability of a strong support system, including family, friends, and healthcare professionals, was found to influence patients’ decision-making process [36]. Factors such as lower work/home responsibilities, travel distance to healthcare facilities, treatment schedules, and recovery time influenced, not significantly, however, BR decisions [24].

How a patient perceives their body post-mastectomy can significantly impact their decision about BR [19,23,33,35,38,40,41]. Some may feel more comfortable and confident with a reconstructed breast [19,21,40,41,45,49], while others may accept their body without BR, especially in cases of not receiving an IBR, as time goes by after diagnosis and treatment, and acceptance replaces mutilation fear [36]. Emotional concerns, such as the need to feel normal and whole again and the need to wear clothes that fit well without prostheses, were mentioned mainly by younger women who chose BR and rated sexuality as important [21,23,38,39,40,41,45,49]. Therefore, regaining psychological empowerment can be a strong incentive underlying the BR choice. Ng et al. [40] and Nair et al. [22] reported that self-confidence improvement is a major factor in BR choices. Psychological distress, meanwhile, is a dominant reason for those unable to consider BR, as cancer diagnosis and the following procedures are already overwhelming [35]. Breast reconstruction can be seen as a step toward emotional healing and recovery from the traumatic experience of breast cancer and mastectomy [2]. Consequently, as Nozawa et al. observed, the decision and process of restoring one’s original physical appearance to the greatest extent possible require a strong sense of control over cancer. This sense of control may act either as a prerequisite—enhancing confidence in coping strategies—or as an outcome, enabling individuals to choose BR and experience its positive effects on QoL [35]. Women who decided not to have BR had a lower level of body image interest due to worries about future complications and additional hospitalizations, concluding that they felt that they did not need it [24,45,49], as Flitcroft et al. also reported in their systematic review [6].

Perception of cancer and fear of recurrence is an ambiguous factor since it has been presented as a determinant in refusing BR, as well as motivating the BR choice to overcome the fear of recurrence, in the sense that the woman has overcome cancer and can move on with her life [39,40,66]. There is confusion and misinformation among breast cancer patients, with many expressing their concern that BR might complicate or delay the detection of recurrence, while others report that BR reduces the risk of cancer in the future by preventing recurrence [9,44]. Patients’ perceptions of cancer, treatment, and recovery can influence their decisions. Some patients may prioritize cancer treatment and recovery over cosmetic outcomes. Women may have different expectations and goals regarding post-mastectomy appearance and physical comfort. BR allows patients to align their physical appearance with their personal goals.

The findings of this systematic review underline the multidimensional nature of BR decision-making, shaped by medical, socioeconomic, and psychological factors. Objective determinants, such as age, financial status, insurance coverage, and physician recommendations, significantly influence BR uptake. Younger women and those with private insurance or higher socioeconomic standing are more likely to opt for BR, while financial constraints, lack of access to specialized surgeons, and regional disparities in healthcare policies pose significant barriers. Additionally, subjective determinants, including body image perception, self-esteem, fear of recurrence, and personal preferences, play a crucial role in shaping BR choices. Women who place high importance on their physical appearance and psychological well-being are more inclined toward BR, whereas concerns about surgical risks and additional procedures discourage others.

Furthermore, healthcare systems considerably impact BR access and decision-making. Countries with universal healthcare models report fewer financial obstacles; however, disparities in awareness, physician recommendations, and patient involvement remain prevalent. In contrast, privatized healthcare systems introduce economic barriers that disproportionately affect lower-income women. Additionally, limited access to BR information—particularly among older women, non-native speakers, and socioeconomically disadvantaged groups—further exacerbates inequities in BR uptake. The role of surgeons and decision-making tools is critical in bridging these gaps, as evidence suggests that structured counseling and SDM models enhance patient knowledge and confidence in BR choices.

Given these complexities, future research should focus on refining SDM models, addressing healthcare provider biases, and ensuring that BR information is accessible to all patients, regardless of age, socioeconomic status, or cultural background. Additionally, longitudinal studies are needed to assess how BR decisions evolve over time and whether patient satisfaction with their choices changes in the years following mastectomy. Healthcare systems can better support women in making informed choices that align with their medical needs and personal values by adopting a holistic approach to BR counseling and decision-making.

### Study Limitations

This review had some limitations. First, the included studies utilized different research approaches, such as surveys, retrospective analyses, and qualitative methods. Since many of these were cross-sectional studies, they captured only a moment in time, making it difficult to assess how BR decisions evolve. Future research that follows patients over time could provide a more comprehensive picture of the decision-making processes.

Moreover, this review primarily included studies published in English, which may have excluded findings from other regions with different healthcare structures and cultural influences. The studies also varied in sample sizes and geographical settings, meaning that the findings may not always be broadly applicable. Some studies were based on national databases with extensive data, while others focused on smaller groups, limiting generalizability.

Another consideration was the age distribution of study participants. Many studies included a higher proportion of older women, which may not fully reflect the perspectives of younger women considering BR. Moreover, while socioeconomic factors were discussed extensively, healthcare access in lower-income European countries and middle-income regions remains an area that requires further exploration, as these settings may have different challenges affecting BR decisions.

Furthermore, self-reported data may have bias, particularly in studies that assess emotional and psychological factors such as body image, self-esteem, and concerns about cancer recurrence. Participants may provide responses influenced by memory recall or social expectations, making it challenging to capture a completely objective view of their motivations.

Additionally, although this review highlighted the influence of surgeons, it did not fully capture the contributions of multidisciplinary teams, such as breast care nurses, psychologists, and oncology social workers, whose roles in BR counseling are increasingly recognized but inconsistently reported across studies.

Finally, while this review considered the role of healthcare providers, it did not fully address broader systemic factors such as insurance policies, healthcare infrastructure, and the availability of reconstructive surgeons. Further studies should investigate these aspects to provide a clearer understanding of how healthcare systems can better support equitable access to BR.

## 5. Conclusions

The decision to undergo BR is influenced by diverse factors, including medical, psychological, socioeconomic, and systemic elements. This review illustrated how age, financial status, healthcare infrastructure, cultural attitudes, and physician guidance collectively shape BR uptake among women worldwide. While individuals with greater financial resources, stronger awareness, and access to informed healthcare professionals are more likely to choose BR, challenges such as economic limitations, lack of accessible information, and biases in medical recommendations continue to hinder equitable decision-making, particularly for older women and those from lower socioeconomic backgrounds.

Enhancing patient-centered BR decision-making requires a standardized and inclusive approach to counseling, integrating SDM models, and expanding educational resources to ensure that all women receive comprehensive, unbiased, and accessible information about their options. Addressing disparities across healthcare systems necessitates policy reforms, targeted physician training, and broader public health initiatives to facilitate informed choices and equitable access to BR services. Future research should focus on long-term patient satisfaction, the psychological effects of BR decisions, and advancements in reconstructive techniques to further refine patient care and outcomes.

Ultimately, supporting women in making informed BR decisions demands an inclusive, well-structured healthcare approach that aligns with their medical needs, personal preferences, and overall well-being.

## Data Availability

No new data were created.

## Supplementary Materials

The following supporting information can be downloaded at https://www.mdpi.com/article/10.3390/healthcare13111307/s1. Table S1: Overview of the selected studies; Table S2: Clinical information; Table S3: Scoring of selected studies according to QuADS criteria; Table S4: Objective factors influencing BR choice; Table S5: Subjective factors influencing BR choice.

## Abbreviations

The following abbreviations were used in this manuscript:

BR	Breast reconstruction
SDM	Shared decision-making
QuADS	Quality Appraisal for Diverse Studies
PRISMA	Preferred Reporting Items for Systematic Reviews and Meta-Analyses
QoL	Quality of life
IBR	Immediate breast reconstruction
PMRT	Post-mastectomy radiation therapy
DBR	Delayed breast reconstruction
SES	Socioeconomic status
nBR	No breast reconstruction
